# Safety and efficacy of single insertion accelerated MR-image guided brachytherapy following chemo–radiation in locally advanced cervix cancer: modifying our EMBRACE during the COVID pandemic

**DOI:** 10.1186/s13014-023-02240-5

**Published:** 2023-03-20

**Authors:** Mark J. Stevens, Florence Ko, Judith Martland, Ryan Brown, Linda Bell, John Atyeo, Jackie Yim

**Affiliations:** 1 Department of Radiation Oncology, Northern Sydney Cancer Centre, Royal North Shore Hospital, Level 1 ASB Building, St Leonards, NSW 2065 Australia; 2 Northern Clinical School, University of Sydney, St Leonards, NSW Australia

**Keywords:** Cervix cancer, Coronavirus-2019, EMBRACE-I, EMBRACE-II, IGABT, Locally advanced cervix cancer, LACC, MRI, MR-Image guided adaptive brachytherapy, VMAT

## Abstract

**Background:**

Utero-vaginal brachytherapy (BT) is an irreplaceable care component for the curative treatment of locally advanced cervix cancer (LACC). Magnetic Resonance Imaging (MRI)-image guided adaptive BT (IGABT) using the GYN-GEC-ESTRO EMBRACE guidelines is the international care standard. Usually following chemo–radiation therapy (CRT), IGABT has high proven utility in LACC but requires significant health system resources. Timely access was disrupted by the COVID-19 pandemic which challenged us to re-design our established IGABT care pathway.

**Methods:**

From April 2020 consecutive patients with LACC were enrolled after CRT in a single arm exploratory non-inferiority study of a modified IGABT (mIGABT) protocol. This delivered an iso-effective IGABT dose (39.3 Gy: EQD2: α/β10Gy concept) over a 24-h period during a single overnight hospitalisation.

**Results:**

Fourteen LACC patients received mIGABT from April 2020 to March 2022. Median age was 62.5 years (37–82 years). LACC histology was primary squamous (9/14) or adeno-carcinoma (5/14). International Federation of Gynaecology and Obstetrics (FIGO) 2018 stages ranged from IB1/2 (N = 3), IIA1/IIB (5), IIIB (2), IIIC1/2 (4) with mean ± standard deviation (SD) gross tumour volume-at-diagnosis (GTV_D) of 37.7 cc ± 71.6 cc. All patients achieved complete metabolic, clinical, and cytologic cancer response with CRT and IGABT. High-risk HPV was cleared by 6-months. Complete MRI-defined cancer response before mIGABT (GTV_Fx1) was seen in 77% of cases (10/13). Only two women developed metastatic disease and one died at 12-months; 13 patients were alive without cancer at mean 20.3 ± 7.2 months follow-up. Actuarial 2-year overall survival was 93%. Compared with our pre-COVID IGABT program, overall mIGABT cost-saving in this cohort was USD 22,866. Prescribed dose covered at least 90% (D90) of the entire cervix and any residual cancer at time of BT (HRCTV_D90: high-risk clinical target volume) with 3-fractions of 8.5 Gy delivered over 24-h (22.8 ± 1.7 h). Total treatment time including CRT was 38 days. The mIGABT schedule was well tolerated and the entire cohort met EMBRACE recommended (EQD2: α/β10Gy) combined HRCTV_D90 coverage of 87.5 ± 3.7 Gy. Similarly, organ-at-risk (OAR) median: interquartile range D2cc constraints (EQD2: α/β3Gy) were EMBRACE compliant: bladder (65.9 Gy: 58.4–72.5 Gy), rectum (59.1 Gy: 55.7–61.8 Gy), and sigmoid colon (54.6 Gy: 50.3–58.9 Gy). ICRU recto-vaginal point dose was significantly higher (75.7 Gy) in our only case of severe (G4) pelvic toxicity.

**Conclusions:**

This study demonstrated the utility of mIGABT and VMAT CRT in a small cohort with LACC. Loco-regional control was achieved in all cases with minimal emergent toxicity. Single insertion mIGABT was logistically efficient, cost-saving, and patient-centric during the COVID-19 pandemic.

## Background

In 2020 the United Nations International Agency for Research on Cancer ranked invasive cervix cancer 4^th^ after female breast, lung, and colorectal cancer in both annual incidence and mortality with approximately 60% of those diagnosed worldwide (342,000/604,127 women) dying of the disease [1]. Standardised cervix cancer mortality rates were 2.5 times higher in low and middle income countries (LMIC) [2]. Reducing poverty and developing sustainable Public Health initiatives that prioritise primary prevention using high-risk Human Papillomavirus (hr-HPV) vaccination [3, 4], and secondary surveillance strategies, are exigent to improving global outcomes as most patients present with locally advanced (inoperable) cervix cancer (LACC) [1–5].

LACC demands reliable access to integrated external beam chemo–radiation therapy (CRT) and utero-vaginal brachytherapy (UVBT) for cure [4–15]. UVBT is deemed an “irreplaceable” or critical component of care [16, 17], but UVBT is often non-existent, difficult to access, or without fidelity in many LMIC [2, 4, 6]. Underuse of UVBT is a major determinant for poorer cervix cancer treatment outcomes in all economic settings [2, 4, 18].

In March 2020, the novel Coronavirus disease 2019 (COVID-19) pandemic acutely disrupted cancer services in high income countries such as Australia. Access delays to UVBT imposed by the pandemic temporally mimicked some of the global realities of LACC in our practice [19–25].

In 2008, the Gynaecological Groupe Européen de Curiethérapie—European Society for Radiotherapy and Oncology (GYN GEC-ESTRO) began the prospective International study of image guided intensity modulated External beam radiotherapy and Magnetic Resonance Imaging (MRI) based adaptive Brachytherapy in locally Advanced Cervical cancer (EMBRACE-I) study. EMBRACE-I proposed multi-fraction 4-dimensional MRI-image guided adaptive brachytherapy (IGABT) after CRT [26] according to institutional tradition. This MRI volume-based prescription, recording, and reporting method has become the consensus international brachytherapy care standard in LACC [27].

Our EMBRACE-I IGABT protocol however, was logistically complex and resource intensive. From inception in 2010, our EMBRACE programme [28] was hospital-based and included 2 overnight admissions separated by 7 days modelled on Vienna dose-fractionation [29]. Each in-patient event involved operating room (OR) access and general anesthesia (GA) for ultrasound-guided utero-vaginal applicator insertion and specialised patient-applicator immobilisation. Subsequent transfers throughout the hospital for daily MRI was a precursor to applicator reconstruction, anatomic contouring, dose optimisation and treatment. Contingencies at any level of this repeated process were nominal.

The COVID-19 pandemic imposed sudden access barriers to protect critical Public Hospital infrastructure and key health personnel. Intra-hospital and Inter-departmental patient transfer restrictions to radiology and radiation oncology facilities further disrupted our EMBRACE care pathway.

Faced with these challenges, we developed a pragmatic radio-biological modification of our institutional EMBRACE protocol. Modelled from a compendium of published COVID-19 fractionation options [30–32] and a small prospective series [33], a putative bio-equivalent accelerated single insertion 3-fraction modified IGABT (mIGABT) EMBRACE programme was trialled on 14 women with LACC. We report our 2-year progression-free survival (PFS), total cost-saving, and toxicity outcomes.

## Methods

This was a single-arm pre-defined exploratory non-inferiority study conducted on 14 consecutive women. All were referred from April 2020 to end-March 2022 with new biopsy-proven HPV-associated LACC. Age < 18 years, cervical neuroendocrine cancer, visceral metastatic disease, and any absolute contraindication to MRI were the only exclusions. COVID-19 post-nasal and throat swabs were polymerase chain reaction (PCR) negative before hospital admission for brachytherapy.

All patients were examined under anaesthesia (EUA) by a radiation oncologist (MJS) and certified gynaecologic oncologist. Speculum findings and the 3-D clinical (palpable) impression of the gross tumour volume (GTV) were recorded. Mandatory radiologic staging included whole body ^18^Fluoro-deoxyglucose (FDG) Positron Emission Tomography/Computer Tomography (PET/CT) and pelvic 3 T-MRI. Total gross primary tumour volume at diagnosis (GTV_D) and at time of IGABT fraction 1 and 2 (GTV_Fx1 and GTV_Fx2) were derived from T2-weighted 3 T-MRI sequences [14].

The decision to recommend definitive CRT/IGABT was determined by our institutional GYN multi-disciplinary team which convened weekly. The design and rationale of our COVID modified EMBRACE IGABT high dose-rate (HDR) protocol, and the analysis of (de-identified) patient databases was pre-approved by the Northern Sydney Local Health District HREC [LNR/2021/ETH01361].

Demographic characteristics, cancer histologic subtype, and International Federation of Gynaecology and Obstetrics (FIGO) 2018 stage [34] GTV_D, and duration of follow-up of our cohort are summarized in Table 1.

**Table 1:** Patient demographics, histology, staging, dose and duration of follow up

Patient	Age (years.)	Histology	Stage (FIGO)	GTV_D	Follow-up (mo.)
1	64	SCC	IIIB	114	30
2	50	SCC	IIIC2	266.2	30
3	58	SCC	IIIC 1	21	29
4	82	SCC	IIB	29.1	26
5	71	SCC	IIIB	23.3	12
6	37	AC(C)	IIIC 1	9.2	25
7	58	AC(C)	IB2	5.5	22
8	48	AC(C)	IIA	7.5	21
9	75	AC(C)	IIB	10.1	16
10	70	SCC	IB1	2.1	15
11	66	AC(A)	IB1	10.2	14
12	61	SCC	IIB	37.4	14
13	46	SCC	IIB	8.4	13
14	66	SCC	IIIC 1	23.1	9

*AC(A)* Adenocarcinoma Silva pattern A; *AC(C)* Adenocarcinoma Silva pattern C; *SCC* Squamous cell carcinoma; *FIGO* Federation of Gynaecology and Obstetrics; *GTV_D* Gross tumour volume-at-diagnosis

### CRT

**Fig. 1:**
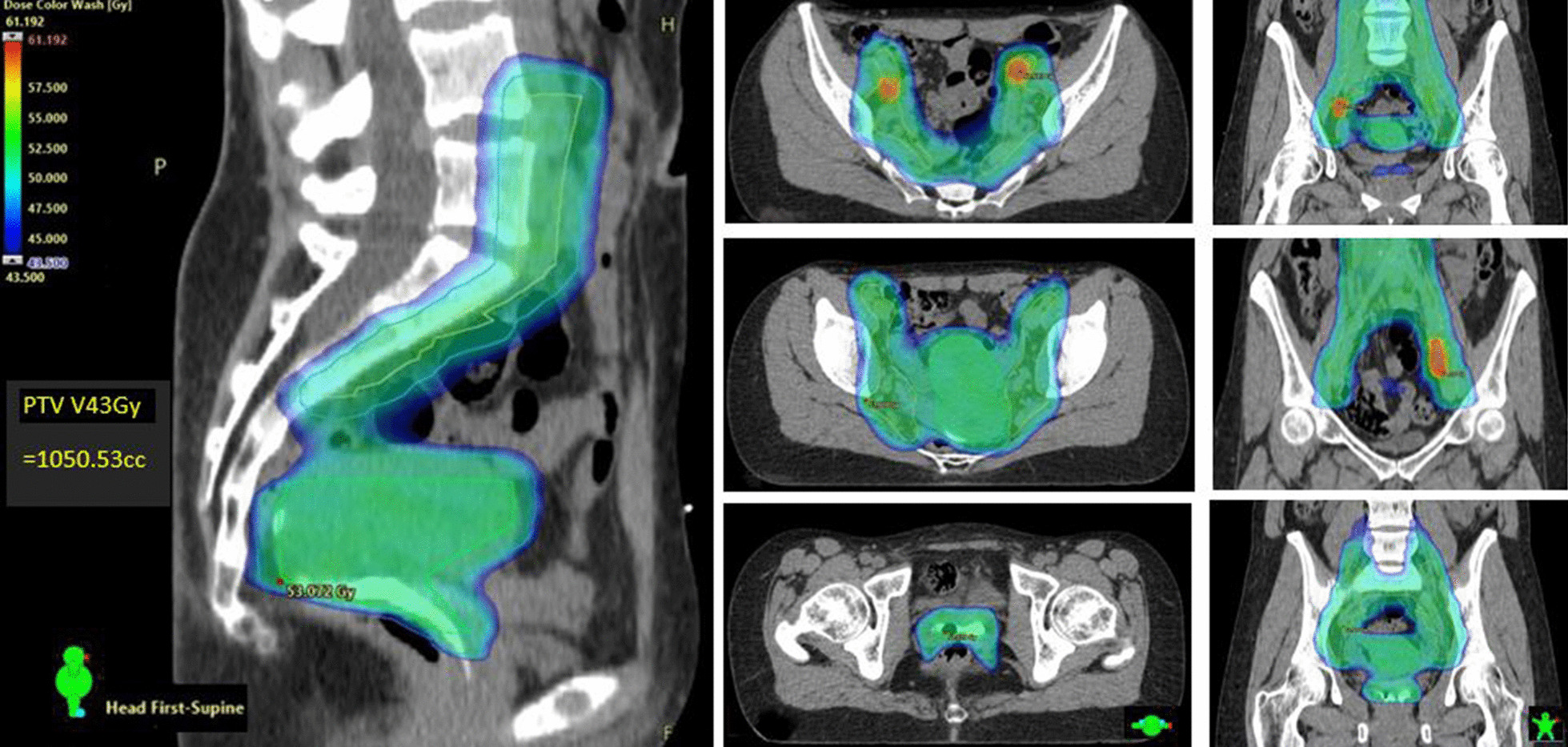
EBRT axial and central sagittal dose-wash showing GTV_N SIB 60 Gy (60 Gy in Red; 52.5–55 Gy in Green; 45 Gy in Blue): note aqueous vaginal contrast

Concurrent 40 mg/m^2^ intravenous cisplatin (CDDP) was prescribed weekly for maximum 5 cycles. External beam radiation therapy (EBRT) was delivered by dynamic single isocentre volumetric-modulated arc therapy (VMAT) on either Varian TrueBeam^®^ or Halcyon^®^ linear accelerators (Varian Medical Systems: Palo Alto, CA). Daily cone-beam computer tomography (CBCT) provided image guidance. The primary central pelvic planning target volume (PTV_P) included cervix and corpus uteri, parametria, and additional vaginal margin on any gross disease identified on staging MRI. Iodated hydroxyethyl cellulose vaginal contrast (Iopromide 5 cc and K–Y gel^®^ 30 cc agitated in 60 cc catheter tip syringe) was routinely used to delineate the vagina from fornix to introitus during simulation (seen fully in Fig. 1 sagittal view). Figure 1 presents the typical EBRT dose wash in axial and sagittal planes. PTV_P and subclinical iliac/para-aortic lymph node disease (PTV_N) was dosed to 45-50 Gy with simultaneous integrated boost to FDG or MRI-defined gross tumour volume nodal metastases (GTV_N; 55-60 Gy) in 25 daily fractions (1.8–2.4 Gy/day). PTVs were expanded by 0.7 cm from primary and nodal clinical target volumes (CTV_P and CTV_N), respectively [7]. Strict bladder (empty) and rectal filling guidelines with daily CBCT verification mitigated uncertainties of internal CTV_P motion. The one patient with FIGO Stage IIIC2 SCC and para-aortic nodal (PAN) metastases had extended field VMAT to the level of the renal vessels. All target volumes and organ-at-risk (OAR) structures were curated according to American Association of Physicists in Medicine nomenclature Task Group 263 [35].

CRT was quality monitored within a collaborative Public–Private Hospital network. All mIGABT was performed within a single Public institution by the same care team.

### Utero-vaginal mIGABT

**Fig. 2:**
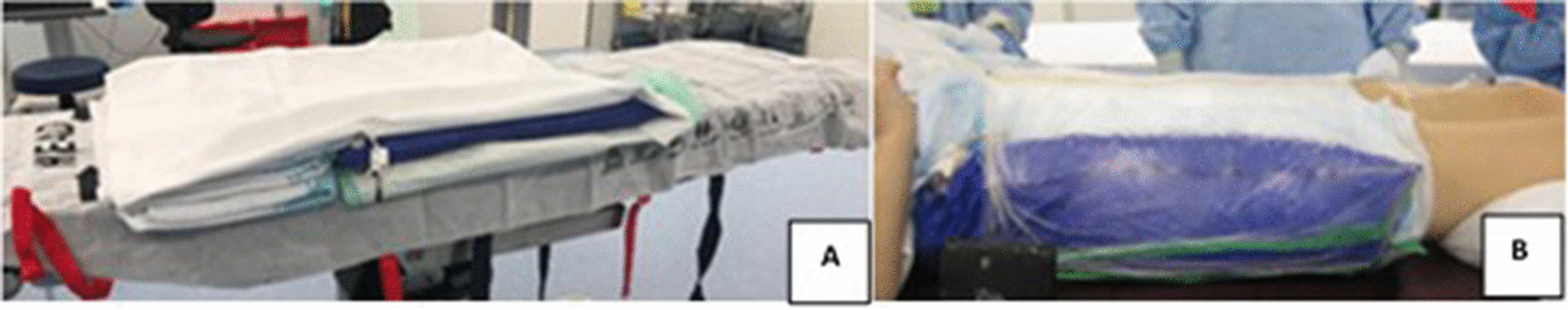
Patient set up **A** Haines^®^ Air-Assisted Transfer Mat set-up **B** Op-site^®^ surgical body wrap securing patient to the BlueBAG™

Day-of-admission utero-vaginal applicator insertion was performed under GA. Specific COVID-19 GA procedures were protocoled to minimise nasopharyngeal aerosolisation, viral dissemination, and unnecessary contacts within the OR [19, 20, 25]. The operating table was pre-prepared with a single use Haines^®^ Air-Assisted Transfer Mat (Haines Medical Australia, manufacturers of the Haines^®^ Air-Assisted Transfer Mat) and reusable vacuum cushion (BlueBAG™; Elekta Solutions AB, Stockholm, Sweden) (Fig. 2A). Applicators were inserted under ultrasound guidance by the radiation oncologist (MJS). Cranio-caudal migration or rotation was prevented by meticulous vaginal (vas gauze) packing and vulvar suturing. Op-site^®^ surgical body wrap secured the patient to the BlueBAG™ (Fig. 2B) which when evacuated formed a rigid cradle from breast-line to upper-thighs. Free draining in-dwelling urinary and rectal catheters were mandatory.

This set-up had been previously validated by us in over 150 LACC patients (> 600 IGABT fractions) since 2010. Significant applicator displacement or pelvic shift during multiple patient transfers was minimal [28]. Immobilisation was well tolerated overnight. Patient-controlled opioid analgesia was routine. A total of six transfer cycles from bed to MRI (pre-Fractions 1 and 2), and CT (pre-Fraction 3) couch top were required to complete mIGABT.

MRI/CT compatible applicators were of 2 types: Vienna Ring Set 26–34 mm (Elekta AB: Part No. 189.569) with fixed angle (60°) intrauterine 20-60 mm tandems (Tandem + Ring (T + R); 12/14 women: 86%), or Vaginal Applicator set (Elekta AB: Part No. 101001): 20-80 mm angled tandems (10–45°) and segmented (20-35 mm) cylinder set (Tandem + Cylinder (T + C); 2/14: 14%). No patient required a hybrid interstitial technique.

**Fig. 3:**
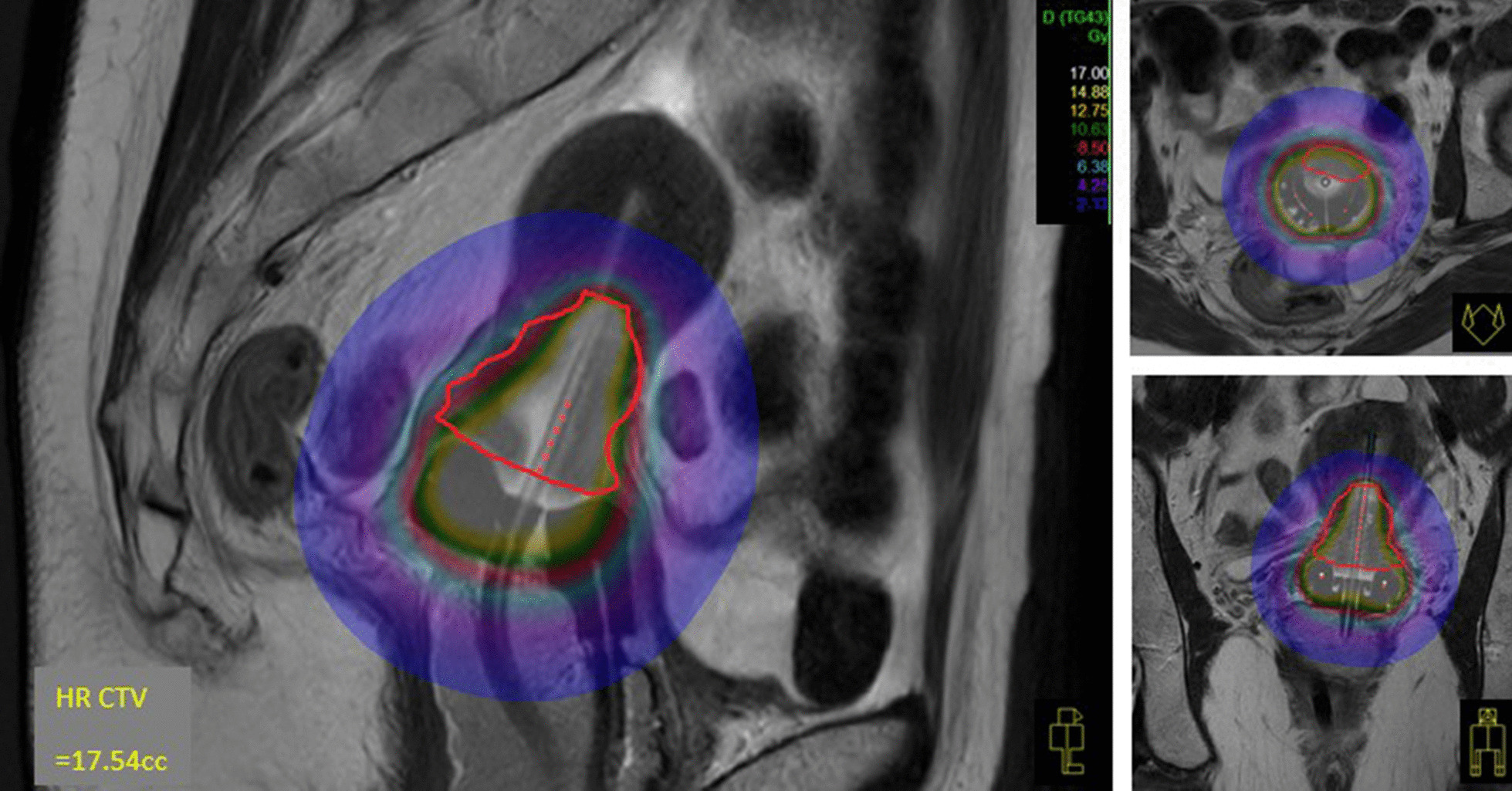
mIGABT (HRCTV in red solid line; 8.5 Gy in red shade). Note hyper-intense tandem and ring central source channels due to oil-filled dummy catheters

A dedicated brachytherapy planning system (Oncentra v4.6.0; Elekta AB) was used to contour, reconstruct, optimise, and evaluate dose target coverage. A standard loading pattern similar to a Manchester “Point A” plan was used as the starting point before manual conservative optimisation to ensure 3D-volume-based planning metrics were met. At least 90% of the ICRU 89 high-risk CTV (HRCTV) received 100% of the prescription dose (HRCTV_D90 concept) (Fig. 3). Pre-specified ICRU 89 OAR D2cc constraints (rectum, bladder, and sigmoid colon) were respected. OAR D0.1 cc and D1.0 cc exposures were also recorded. Emergent ICRU rectal, bladder, and vaginal late toxicity dose points, and vaginal reference length (VRL) were also identified [32, 36]. Vaginal toxicity points were located 0.5 cm deep to the mucosa in the anterior and lateral fornices, and for T + R patients (12/14), relative (± 2 cm) to the postero-inferior border of symphysis pubis (PIBS). The VRL was defined longitudinally from the PIBS point to the centre of the Vienna ring (vaginal sources).

### MRI/MRI and MRI/CT image match

Fraction 1 HRCTV_D90 and OAR volumes were defined on the day of applicator insertion on T2-MRI sequences (Ingenia 3.0 T CX: Philips Medical Systems, Eindhoven, Netherlands), and imported into Velocity^®^ (v4.1, Varian Medical Systems: Palo Alto, CA). Velocity^®^ allowed rigid applicator registration and assessment of dosimetric uncertainties due to applicator displacement after Fraction 1. Oil-filled markers were placed within the applicators pre-imaging to aide localisation (Fig. 3). Relative inter-fractional applicator shift on Fraction 2 and Fraction 3 was primarily assessed by tandem tip/flange position (all cases), and ring (12/14) or cylinder dome (2/14) tilt, yaw, and migration. Secondary match structures included posterior bladder (empty) wall, rectal wall/tube, and symphysis pubis. Overall 3-D stability (± 1–2 mm) was confirmed using the Velocity^®^ platform before Fractions 2 and 3. Based on internal departmental analysis and Schindel et al. [37], a tolerance of ≥ 3 mm was applied to the matching structures before brachytherapy re-plan was required.

### mIGABT accelerated hyper-fractionation

Modified IGABT was delivered with HDR ^192^Ir (microSelectron^®^ v3.1.4, Elekta AB). The EMBRACE/ICRU 89 IGABT protocol delivered a biologically-equivalent tumour dose (Linear-Quadratic [LQ] model: EQD2 α/β10Gy) of 39.7 Gy (4 IGABT fractions of 7 Gy in 2 insertions to the HRCTV_D90 over 8 days). We hypothesised a bio-effective mIGABT scheme to be 39.3 Gy (25.5 Gy in 3 fractions of 8.5 Gy in 1 day) as derived from the formula:

*EQD2 mIGABT* = *Total modified dose (d x number fractions) x (α/β* + *d) (α/β* + *2)*^*−1*^* Gy, where d* = *modified dose (Gy)/fraction, and α/β for acute tumour effect* = *10 Gy. EQD2 exposures for OAR late (injury) effects were derived at α/β* = *3 Gy.*[12]*.*

Repair half-time within the LQ model was assumed to be 1.5 h. Our protocoled minimal overnight fractionation interval (Fx1 and Fx2: 17.9 ± 1.3 h), and day 2 gap (i.e. between Fx2 and Fx3: 5.3 ± 0.8 h), was probably sufficient to disregard any significant repair of sub-lethal injury.

The LQ EQD2 concept enabled direct summation of MRI-image-based dose-volume histogram mIGABT and CRT parameters (total HR-CTV_D90 coverage; EQD2 α/β10Gy). Similarly, total OAR exposures for probability of late toxicity (EQD2 α/β3Gy) were also calculated.

### Toxicity

All acute and emergent (> 90 day) late toxicity data was collected by physician and patient-reported assessments according to the Common Terminology Criteria for Adverse Events (CTCAE) v4.03 [38]. Anatomically, five at-risk CTCAE system organ classes (SOC) were identified namely, 1. Blood and lymphatic system, 2. Gastrointestinal, 3. Renal and urinary, 4. Reproductive system, and 5. Musculoskeletal/connective tissue (insufficiency fractures). Specific SOC-related blood tests (full blood count and renal function) were administered during quarterly follow-up together with bi-manual pelvic examination, cervical liquid-based cytology and HPV “co-test” (Cobas^®^ 4800, Branchburg NJ: Roche Molecular Systems, Inc; 2018). Re-staging FDG-PET imaging was done at 3 months, 12 months, and 24 months post UVBT. Diagnostic MRI, FDG-PET, and directed re-biopsies were repeated at any time if clinically indicated.

### Cost analysis

In addition to reporting toxicity outcomes, a cost analysis was conducted to compare the cost of delivering mIGABT versus IGABT. This analysis was conducted with a health service perspective using a bottom-up approach. Labour costs were calculated based on NSW Health Awards [39–42] and the time that it took per task. In-hospital costs were based on departmental figures. All costs are reported in 2021 Australian dollars (AUD/0.72USD).

### Statistical analysis

Descriptive and frequency statistics were used to delineate mean ± SD, and median with interquartile range (IQR) for non-normal data. Scatter plots depicted relationships between dose, volume, OAR point and linear vaginal parameters.

## Results

Table 1 presents patient demographics for the 14 patients included in the study. Median age of our cohort was 62.5 years (37–82 years). Nine women (64%) had primary squamous cell cancer (SCC) and 5 (36%) had endocervical adenocarcinoma (AC). Four patients (29%) had lymph node metastases; pelvis only (Stage IIIC1; 3 cases), and 1 case (Stage IIIC2) with involvement of both pelvic and para-aortic nodal stations. The five AC cases were further classified as either Silva Pattern A (1/5) or unfavourable Pattern C (4/5) [43]. Mean follow-up was 20.3 ± 7.2 months. No patient had acquired COVID-19 infection when PCR tested on day of admission for mIGABT.

All patients completed both radiation components (EBRT and mIGABT). Mean EBRT PTV dose was 46.3 ± 2.7 Gy.

Patient 11 with favourable AC (Silva Pattern A) and minimal residual GTV_D (below) had mIGABT upfront as COVID-19 restrictions delayed her CRT. This patient also discontinued cisplatin (CDDP) chemotherapy after 3 cycles due to fatigue; 5 planned cycles of weekly CDDP were otherwise completed by the other 13 patients.

Total time to complete treatment (CRT + mIGABT) was exactly 38 days. Mean overall mIGABT treatment time was 22.8 ± 1.71 h.

### Volumetric response

Mean cervix gross tumour volume-at-diagnosis (GTV_D) was 37.7 ± 71.6 cc. Excluding Patient 2 with very bulky central disease (266.2 cc), the mean GTV_D of our cohort was 20.2 ± 29.5 cc. Diagnostic loop excision of transformation zone was performed on both Stage IB1 patients (Patient 10 and Patient 11). This resulted in minimal residual disease before treatment (GTV_D: 0.2 cc and 2.1 cc respectively).

Respective median (IQR) HRCTV were Fraction 1: 18.6 cc (16.4–21.7), Fraction 2: 17.9 cc (16.0–21.8), and Fraction 3: 17.8 cc (14.4–22.2). Mean ± SD mIGABT HRCTV_D90 [EQD2: α/β10Gy] coverage was 37.5 ± 3.5 Gy. Combined total mean ± SD and median (IQR) HRCTV_D90 coverage was 87.5 ± 3.7 Gy and 89.4 Gy (87.2–89.6), respectively.

Complete MRI-defined cancer response before mIGABT (GTV_Fx1) was seen in 77% of cases (10/13). Measurable disease at fraction 2 was detected in 4 cases (GTV_Fx2): Patient 2 (21 cc), 4 (0.34 cc), 5 (4.4 cc), and 8 (1.3 cc). Minimal inter-fractional HRCTV adaption was required.

### Velocity^®^ matching

Dosimetric re-plans were necessary in only 3 patients (2/14: Fraction 2; and 1/14: Fraction 3). Contributors to this low frequency included geometric stability of patient-applicator setup, no or small residual GTV at time of mIGABT (13/14; 93%), and minimal perturbations in OAR volumes over 24 h. The Velocity^®^ platform allowed rigid registration of pelvic simulator CT acquired in the brachytherapy facility and obviated the Fraction 3 MRI simulation and the associated in-patient bed transport to radiology.

### Local control

All patients achieved complete clinical, metabolic (FDG-PET), and cervical “co-test” response at 3 months. High-risk human papilloma virus (hr-HPV) was cleared in every case either at this point (11/14: 79%) or at 6 months follow-up (3/14; 21%). Although Patient 1 and Patient 5 progressed systemically, no significant local symptoms were recorded (patient reported SOC: renal and urinary, and reproductive system), or was detected on re-imaging after salvage chemotherapy (Patient 1). This implied durable LACC control within the pelvis. Two-year actuarial PFS was 86% (12/14) and overall survival 93% (13/14).

### Metastases

Systemic metastatic disease occurred in 2 cases (Patient 1 and Patient 5). Both had unfavourable Stage IIIB SCC with significant vaginal disease. Time-to-metastatic event was 10 and 12 months respectively. Patient 5 developed peritoneal metastases and died of sepsis at 12 months post-mIGABT without further cancer-directed care. Patient 1 was diagnosed with a solitary (biopsy-proven) lung metastasis on routine re-staging FDG-PET at 12 months. Salvage cisplatin/paclitaxel doublet chemotherapy with bevacizumab [44] resulted in complete metabolic (FDG) response which is maintained at 24 months.

### Cost analysis

In this context, the cost of delivering IGABT (28 Gy/4 fractions) is 6,024.98 AUD and 3,756.49 AUD to deliver mIGABT (25.5 Gy/3 fractions). Adopting our mIGABT approach resulted in Public Health cost savings of 2,268.49 AUD (1,633.31 USD) per treated patient (Table 2). To date, we have treated 14 patients with our mIGABT protocol and it has saved our institution 31,758.84 AUD (22,866.38 USD). In a large public teaching hospital such as ours, we treat approximately 15 cases of cervix cancer a year. If mIGABT is delivered standardly to this patient cohort then the total projected amount saved per year will be 34,027.35 AUD (24,949.65 USD).

**Table 2:** Procedure cost for 3 fractions versus 4 fractions

Procedures	Cost (AUD)	Cost (AUD)
Theatre	496.40	992.80
Recover	40.49	80.98
RadOncologist	1459.11	2918.22
Diagnostic (US + MRI)	130.22	260.44
Pain CNC	65.00	130.00
Ward 8F (2 nights)	1488.00	1488.00
Equipment sterilisation	77.27	154.54
*Total cost*	3756.49	6024.98

*AUD* Australian Dollars

### Toxicity

Both components of care (CRT + mIGABT) were well tolerated. Acute cysto-proctitis during CRT was usually minor (≤ G2) and self-limiting in 13/14 (93%). Excluding Patient 2 (extended-field; pelvis + PAN), median pelvis-only PTV V43Gy was 1360 cc (1322–1418.5 cc).

Severe (≥ G3) acute toxicity was documented in Patient 2 only. As previously stated, this case had massive GTV_D (266.2 cc) and was also the only patient who required extended-field VMAT due to gross para-aortic lymph node metastases (Stage IIIC2). Final PTV V43Gy (2407.3 cc) and V55Gy (1117.5 cc) were both significant [7]. Haemorrhagic proctitis was associated with therapeutic anti-coagulation for significant pelvic venous thrombosis and pulmonary embolism at diagnosis. Rectal bleeding settled with transition to fractionated heparin and glucocorticoid suppositories. Patient 2 was also the only case who developed severe late toxicities across all 5 SOC groups, viz., 1. Blood and lymphatic system: anaemia requiring transfusion, 2. Gastro-intestinal tract: recurrent/persistent low volume haematochezia and diarrhoea, 3. Renal and urinary system: haematuria/bilateral ureteric stenosis (unilateral stent), 4. Reproductive: significant vaginal stenosis/vaginismus and dyspareunia, and 5. Musculo-skeletal system: asymptomatic pelvic (sacro-iliac) insufficiency fracture was noted on routine FDG-PET at 12 months. Pelvic examination, cervical co-test, and re-staging FDG-PET at 24 months, however confirmed healed fracture and cervix cancer-free status. Unfortunately, a wide vesico-vaginal fistula developed at 26 months following a stretch vaginoplasty and necessitated an anterior exenteration (G4 late toxicity). No histologic evidence of residual cervix cancer was present within resected tissue.

OAR exposures at completion of treatment were well within ICRU 89 guidelines. Respective total median D2cc. [EQD2α/β3Gy] for bladder, rectum, and sigmoid colon was 65.9 Gy (58.4–72.5), 59.1 Gy (55.7–61.8), and 54.6 Gy (50.3–58.9). Median dose at ICRU dose points for bladder 62.9 Gy (52.3–79.6) and recto-vaginal (RV) septum was 66.2 Gy (53.6–80.7). By comparison, absolute ICRU RV dose in Patient 1 and Patient 2 was 87.8 Gy and 75.7 Gy respectively, after T + C brachytherapy. Table 3 depicts combined HRCTV metrics for individual patients with ICRU Point-A doses for comparison. Most V200 (mean 27 ± 9 cc.) was restricted to the applicator system even in Patient 1 (23 cc.) and Patient 2 (16.5 cc.). Mean (SD) TRAK (total reference air-kerma rate at 1 m) was also acceptable/low at 0.0034 Gy (0.0016) and well within the 0.02 Gy associated with significant late bowel morbidity by Bockel et al. [45].

**Table 3:** Individual final combined target (HRCTV) and ICRU Point A dose with TRAK. Relative volume of HRCTV encompassed by up to twice the combined prescription dose (V200) also depicted

Patient	HRCTV Vol (cc.)	HRCTV D90 + EBRT (Gy)	V100 (%)	V150 (%)	V200 (%)	TRAK (Gy)	Point A (Gy) (mIGABT + EBRT)
1	48.0	73.6	74.9	38.3	23.0	0.00514	64.8
2	68.0	80.3	81.7	27.5	16.5	0.00804	83.4
3	17.0	89.1	89.7	45.5	33.5	0.00306	70.7
4	13.9	84.9	90.7	50.7	28.4	0.00293	58.6
5	22.3	80.3	88.0	59.9	39.8	0.00364	69.8
6	17.5	82.1	86.8	48.4	27.7	0.00326	60.7
7	18.1	89.7	90.9	33.0	11.4	0.00306	60.5
8	21.8	84.6	90.5	48.0	19.5	0.00345	65.7
9	27.7	87.3	90.0	45.7	19.3	0.00322	60.8
10	10.3	84.3	90.6	60.3	40.7	0.00153	55.5
11	8.3	84.6	90.3	54.1	34.5	0.00129	52.8
12	21.0	84.5	90.2	56.2	33.5	0.00349	63.7
13	15.7	84.6	90.3	48.7	19.6	0.00278	56.1
14	13.3	89.8	90.5	55.6	30.1	0.00274	63.9

*HRCTV* High risk clinical target volume; *EBRT* External beam radiation therapy; *TRAK* Total reference air-kerma rate at 1 m; *mIGABT* Modified image guided adaptive brachytherapy

In LACC the vagina is both an OAR and potential target volume. Vaginal ICRU points reflected brachytherapy dose gradients at the level of vaginal sources 5 mm deep to the surface of the vaginal ring (12/14 cases) or cylinder dome (2/14; T + C cases). The exposures are representative at the mid-circumference within the 4 quadrants of the vaginal fornix. The T + C set did not allow differential (anisotropic) dosing and were excluded. Respective IGABT median (IQR) right, left, anterior, and posterior vaginal point exposures (12/14; T + R patients) were: 6.2 Gy (5.6–6.9), 5.8 Gy (4.9–7.1), 4.1 Gy (3.4–4.9), and 5.0 Gy (3.6–6.4). Total absolute doses were 52.5 Gy, 52.1 Gy, 50.4 Gy, and 51.3 Gy, respectively.

**Fig. 4:**
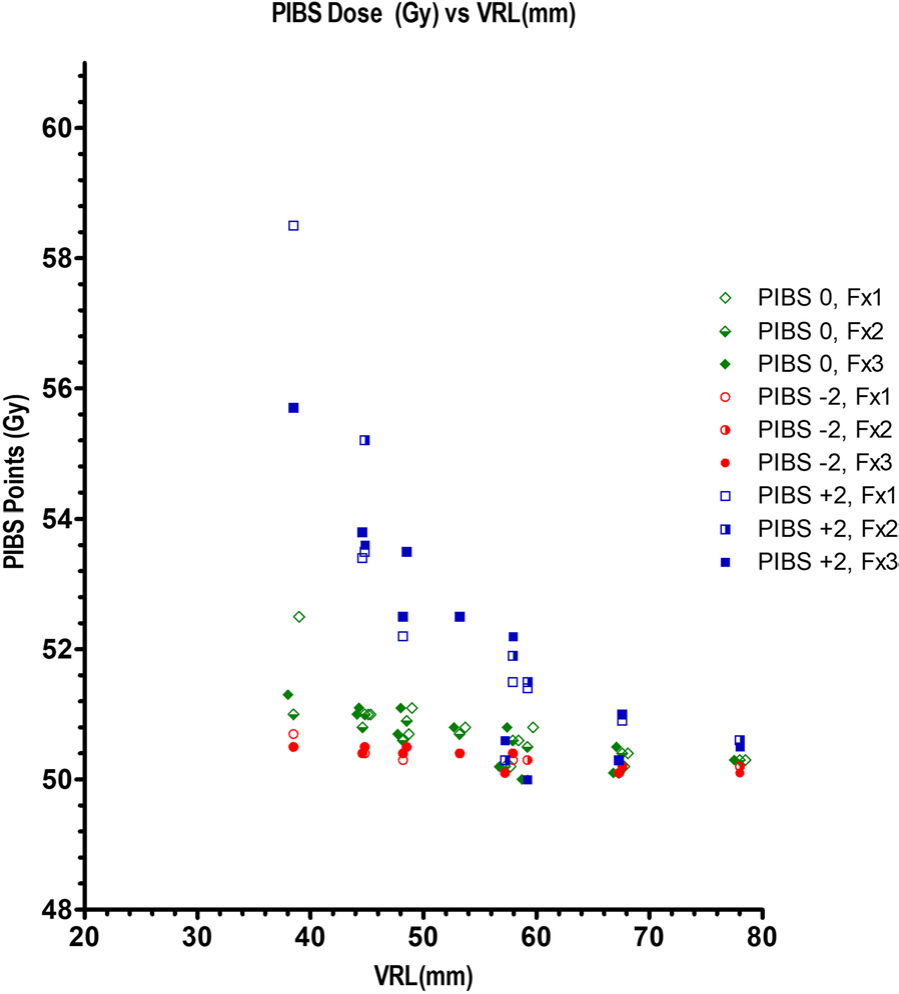
Vaginal OAR data to median total doses administered at PIBS points (PIBS ‘0’ ± 2 cm) versus VRL

Table 4 displays combined dose OAR D2cc exposures for bladder, and rectum versus mIGABT bladder, recto-vaginal (RV), and vaginal ICRU dose point measurements. Figure 4 relates the vaginal OAR data to median total doses administered at PIBS points (PIBS ‘0’ ± 2 cm) versus VRL. By definition VRL could only be derived in the 12 women with T + R applicators; mean ± SD and median (IQR) VRL was 55.4 mm ± 10.7, and 55.2 mm (48.3–60.0) respectively. Predictive modelling for emergent late radiation toxicity is suggested in the Discussion.

**Table 4:** Combined dose OAR D2cc. exposures for rectum and bladder versus mIGABT bladder & recto-vaginal (RV) ICRU dose point measurements

Patient	ICRU RV point total EQD2 (EBRT + mIGABT)	ICRU bladder point total EQD2 (EBRT + mIGABT)	Rectum D2cc total EQD2 (EBRT + mIGABT)	Bladder D2cc total EQD2 (EBRT + mIGABT)
1	77.8	73.2	70.0	67.3
2	80.7	79.6	74.5	89.8
3	71.4	61.4	54.8	65.0
4	65.6	54.4	58.7	66.8
5	71.0	78.0	59.5	89.9
6	58.5	54.0	61.9	41.6
7	71.9	62.5	58.7	68.7
8	65.5	70.7	62.5	73.8
9	66.2	64.2	61.5	79.5
10	53.6	52.3	48.5	50.9
11	53.4	57.0	45.2	52.8
12	70.7	63.3	58.5	57.1
13	61.2	58.9	54.1	62.2
14	66.2	67.0	61.2	63.7

*ICRU RV* International Commission of Radiation Units Recto-Vaginal; *EQD2* Equivalent dose in 2 Gy fractions; *EBRT* External beam radiation therapy; *mIGABT* Modified image guided adaptive brachytherapy

## Discussion

Over a 15-year period a landmark series of Gynaecological (GYN) GEC-ESTRO Working Group Recommendations (I–IV) described a coherent methodology of utero-vaginal MRI-IGABT prescription, recording, and reporting in LACC [11–14]. These publications (2005–2012) introduced the concepts of GTV and high-risk clinical target volume (HRCTV), and OARs in the familiar epistemology of 3D-EBRT. Similar to EBRT, brachytherapy dose became tractable as bio-equivalent daily fractions of 2-Gray based on differential tissue repair kinetics (Linear Quadratic EQD2Gy model) [12]. Personalised target and OAR dose-volume parameters had replaced the historical anatomic and prescription reference points of the traditional 2D-cervix brachytherapy traditions (or “Schools”).

Based on these principles, and through prior IGABT clinical practice [46, 47], the GYN GEC-ESTRO international EMBRACE-I study (2008–2015; 1416 patients) produced “unprecedented” actuarial 5-year rates of cervix cancer control (92%; 1233/1341 analysed). Rates of serious (≥ G3) OAR late toxicity were low (cumulative 14.6%; 3–6% per organ system). Remarkably, compared with mono-institutional IGABT cohorts [44, 47], the 5-year overall survival improved even in advanced Stage IIIB/IVA patients (74% to 79%). Historical 5-year disease-specific survival rates of only 35% [48] were expected in Stage IIIB women often due to suboptimal local and regional nodal control. Sustained eradication of central pelvic and nodal metastatic disease in EMBRACE-I however, were both excellent (87%) at 5-years. The significant predictive impact of pelvic and nodal control on overall survival in patients with LACC were confirmed in a recent nomogram from the GYN GEC-ESTRO group [47].

EMBRACE-I established the combined (EBRT/IGABT) minimal HRCTV_D90 target dose of 85 Gy [EQD2: α/β10Gy] as a hard goal for controlling the central pelvis. A range of safe OAR D2cc exposures (EQD2: α/β3Gy) were also reported. These have since been refined as primary planning aims in the current EMBRACE-II study [15]. Specifically, bladder D2cc (< 80 Gy), rectum D2cc (< 65 Gy), and sigmoid/bowel D2cc (< 70 Gy) were recommended constraints in addition to maximal ICRU recto-vaginal (RV) dose point exposure of less than 65 Gy. The latter correlates with vaginal shortening and stenosis [32, 36]. The principles and parameters of EMBRACE-I were validated by ICRU Report 89 [27]. ICRU 89 (2016) remains an enduring global capstone in the healthcare of women with LACC.

Despite these gains, optimal treatment of LACC is technically complex and health resource consumptive. In LMICs with the highest prevalence of cervix cancer, sophisticated oncologic care is simply unavailable or difficult to access [2, 4, 6]. Furthermore, over 90% of MRI scanners exist in high-income-countries. Democratising LACC treatment would require major LACC treatment re-design at several levels beginning perhaps with point-of-care enhanced low static field (LF) MRI units [49]. LF-MRI (0.25–1.0T) does not require expensive cryogenics, and has modest energy consumption, installation, and maintenance costs. Compact open-system LF-MRI configurations are ideal for MRI “fluoroscopy” and real-time (metal) applicator tracking for volume-based IGABT in remote areas. With appropriate industry and academic partnerships, a sustainable locally managed single insertion IGABT programme could be feasibly implemented in some LMIC settings [2, 4, 6].

Although major healthcare disparities are rare in Australia [50], the 2020 COVID-19 pandemic introduced uncertainties in patient care and sudden access delay to Public Health infrastructure. This challenged us to modify our original Vienna 4-fraction EMBRACE protocol [26, 29]. Re-design to an iso-effective single insertion IGABT protocol immediately halved our operating room requirements and in-patient transfers for MRI scans. The economic cost of our mIGABT schedule was shown with net savings of 2,268.49AUD (1,634USD) per treated patient (Table 2). There were no acute (≤ 3 months) re-hospitalisations.

This non-inferiority study of 14 women with LACC demonstrated the real-world “fungibility” of the radio-biological backbone of EMBRACE-I. Our IGABT fraction sizes of 8.5 Gy via a single utero-vaginal insertion delivered a mean (SD) combined HRCTV_D90 of 87.5 ± 3.7 Gy (EQD2Gy) in 3 fractions accelerated over 24 h. This schedule with pre-emptive or sequential CRT was generally well tolerated. All indices of HRCTV_D90 coverage, OAR, and most individual organ system ICRU dose point exposures were within published efficacy and chronic safety recommendations. Overall treatment time was significantly less than 50 days (38 days). Actuarial overall metabolic (FDG-PET) progression-free survival was 93%. All patients achieved central pelvis control with early eradication of malignant cytology (3-months) and hr-HPV (by 6-months) on PCR co-testing. Lymph node failure or persistent nodal metastatic disease did not occur. Our EBRT induction platform of dynamic VMAT and simultaneous accelerated integrated boost (55-60 Gy) to GTV_N with full chemotherapy intensity in 93% of patients (13/14) is assumed to have contributed to this success [7, 8, 32]. Registration of the pre-treatment MRI/FDG-PET to the pelvic CT-simulation series aided localisation of GTV_N. Rules for CTV_P and CTV_N contouring and OAR delineation were consistently applied since first adopting EMBRACE-I standards in 2010.

Our experience paralleled that of Mahantshetty et al. [33]. Their Phase I/II SIMBRACE trial utilised a single insertion 3-fraction MRI-IGABT regimen with HRCTV_D90 coverage of 9 Gy (Day 1) and 2 fractions of 7 Gy separated by 6-h on Day 2. Inter-fractional CT imaging determined the requirement for dosimetric re-plan. SIMBRACE had mean overall treatment time 9 days longer at 47 ± 6 days and their EBRT platform doses was higher (50 Gy) ± weekly CDDP chemotherapy. Two-year pelvic control was 90.1% in FIGO IIB-IVA patients with a comparable low rate (5.3%) of G3 rectal toxicity in the 38 women treated on protocol.

Although a post-COVID-19 change in our pattern of brachytherapy practice awaits longer follow-up, limitation to our method was suggested by significant late toxicity signals in one patient (Patient 2) with advanced central pelvic cancer (GTV_D: 266.2 cc). Extensive residual upper and middle third vaginal disease after CRT (HRCTV: 68 cc.) required a less optimal T + C IGABT technique which did not permit differential loading at the cervix level. The final combined ICRU RV point dose was almost 137% higher than the cohort median (75.7 Gy vs. 55.3 Gy) despite a low cancer residuum within the cervix (24 cc). ICRU Point A dosing was also highest (Table 3): 83.4 Gy (remainder cohort mean: 61.8 ± 5.3 Gy) although TRAK was relatively low (0.008 Gy). TRAK (≥ 0.02 Gy), residual HRCTV (> 25 cc.), and prior “active smoking” have been identified as risk factors for emergent bowel toxicity in a large multivariate analysis by Bockel et al. [45]. Although a prior tobacco smoker (10–20 pack-years), her EBRT was well within EMBRACE dose-volume D95 guidelines for extended-field radiation therapy (< 3500 cc): PTV V43Gy (2407.3 cc). Neo-adjuvant cyto-reductive chemotherapy [44] and hybrid (intracavity/interstitial) IGABT [46] were considered but the malignancy-associated coagulopathy with heavy vaginal bleeding, extensive deep venous pelvic thromboses, and significant pulmonary embolism were unique factors complicating this patient’s presentation.

## Conclusions

This small study has demonstrated the utility of single insertion 3-fractional utero-vaginal MRI- IGABT and CRT in a cohort of 14 women with LACC. Loco-regional control was achieved in all cases. Single insertion IGABT was logistically efficient, overall cost-saving, and patient-centric during the COVID-19 pandemic. Severe pelvic toxicity in one patient (Patient 2) who is cancer-free at 30-months post-anterior exenteration, implicated both large residual HRCTV, history of cigarette smoking, and mIGABT applicator type to risk of organ-system injury.

## Data Availability

The datasets used and/or analysed during the current study are available from the corresponding author on reasonable request.

## Abbreviations

3 T: 3 Tesla
AAPM: American Association of Physicists in Medicine
BT: Brachytherapy
CBCT: Cone beam computer tomography
CCR: Combined composite response
CDDP: Cisplatin
COVID-19: Coronavirus disease 2019
CRT: Chemo–radiation therapy
CTCAE: Common Terminology Criteria for Adverse Events
CT: Computer tomography
CTV: Clinical target volume
CTV_N: Clinical target volume nodal
CTV_P: Clinical target volume primary
EBRT: External beam radiation therapy
EMBRACE: Image guided intensity modulated External beam radiotherapy and MRI based adaptive BRAchytherapy in locally advanced CErvical cancer
EQD2: Equivalent total doses in 2 Gy fractions
EUA: Examined under anaesthesia
FDG: Fluorodeoxyglucose
FIGO: International Federation of Gynecology and Obstetrics
GA: General anaesthetic
GTV: Gross tumour volume
GTV_D: Gross tumour volume-at-diagnosis
GTV_N: Gross tumour volume nodal
GYN GEC-ESTRO: Gynaecological Groupe Européen de Curiethérapie -European Society for Radiotherapy and Oncology
HDRB: High dose-rate brachytherapy
HIC: High income countries
hr-HPV: High risk human papillomavirus
IARC: International Agency for Research on Cancer
ICRU: International Commission on Radiation Units and Measurements
IGABT: MRI-guided adaptive utero-vaginal brachytherapy
LACC: Locally advanced cervix cancer
LMIC: Low and middle income countries
LQ: Linear quadratic
mIGABT: Modified MRI-guided adaptive brachytherapy
MRI: Magnetic resonance imaging
OAR: Organ-at-risk
OR: Operating room
PAN: Para-aortic nodal
PCR: Polymerase chain reaction
PET: Positron emission tomography
PFS: Progression free survival
PIBS: Postero-inferior border of the symphysis pubis
PTV: Planning target volume
PTV_N: Planning target volume lymph node
PTV_P: Pelvic planning target volume
RV: Recto-vaginal
SD: Standard deviation
SOC: System organ classes
T + C: Tandem + Cylinder
T + R: Tandem + Ring
UVBT: Utero-vaginal brachytherapy
VMAT: Volumetric modulated arc therapy
VRL: Vaginal reference length
